# Standardization of Particle Size for Floating Particle Wettability Measurement for Carbonate Rocks

**DOI:** 10.1021/acsomega.2c06679

**Published:** 2023-03-20

**Authors:** Bisweswar Ghosh, Hadi Belhaj, Huda Alhashmi, Francis Idachaba, Parth Joshi, Md. Motiur Rahman, Mohammed Haroun

**Affiliations:** † Petroleum Engineering, Khalifa University, Abu Dhabi 127788, United Arab Emirates; ‡ University of North Dakota, Grand Forks, North Dakota 58202, Canada; § Schlumberger Ltd., Mr., Gurgaon 77042, India

## Abstract

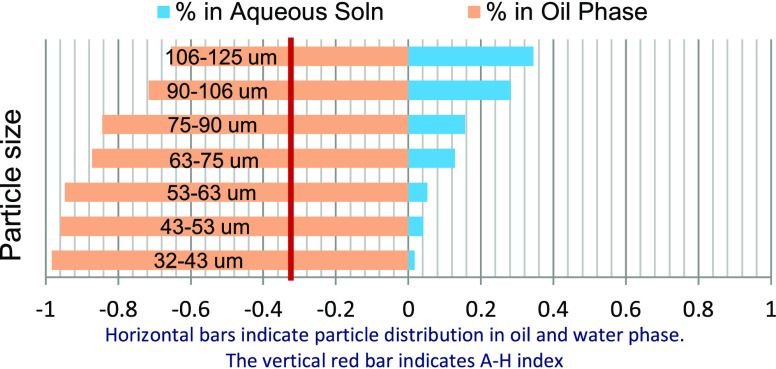

Misrepresentation of the wettability of a reservoir can lead to potentially low ultimate hydrocarbon recovery resulting in substantial economic losses. At the same time, it is impossible to determine the wettability of a reservoir across its length and breadth on a continuous basis using standard procedures. This work presents the development and standardization of a quick, easy, and low-cost wettability measurement method using the adherence tendency of rock particles in the oil or aqueous phase. The most important aspect of this study was establishing the optimum particle size for sustained floatation and balancing the buoyancy and gravity effect. The results show that the particles sink with a larger than optimum particle size because of the gravity effect. Similarly, the particles would float if they are smaller than optimum due to buoyancy and viscosity advantages. A new scale is designed, and the midpoint analysis shows that a 63–90 μm particle size is the ideal size range for the carbonate reservoir’s wettability measurements, as the midpoint of the size distribution coincides with the standard Amott–Harvey (A–H) index. However, this size range is found to be wider for oil-wet particles. The floating particle method has several advantages over the established methods once standardized against a reliable process. Not only is the process fast but it can be performed with basic laboratory tools and does not require a high skill set. Most importantly, reliable wettability information can be obtained from drill cuttings and core fragments, enabling the determination of reservoir wettability on a continuum basis and not as a point basis, thus providing a more reliable average value, particularly for heterogeneous and unconsolidated reservoirs.

## 1 Introduction

The importance of reservoir rock wettability cuts across every oil and gas development phase. It is recognized as a significant factor influencing multiphase flow, heat transfer, solute transport, and synthetic and natural porous media.^1−3^ In the exploration stage, wettability helps to estimate the recoverable hydrocarbon volumes and the life span of the producing wells.^4^ A general trend of the expected dry or virgin hydrocarbon production (without water cut or free gas) can be observed from existing fields and producing formations worldwide. Hence, the wettability of the formation gives a qualitative estimation of the water breakthrough period and the period at which the well will cease to flow. As a result, the wrong assessment of the wettability misleads the expected life span of the development wells. The development stage is also affected by wettability since, most probably, the large pores are occupied by the nonwetting phase fluid, whose movement is easier and faster than the wetting fluid. Thus, field operators prefer to have rocks with a higher affinity toward water to achieve a higher recoverability of oil.

The enhanced oil recovery comes after the primary and secondary recovery stages. It is well known that EOR requires a lengthy screening process to decide on the optimum production methodology. Wettability comes with great significance in this stage, where a variety of options and processes are discussed, such as using surfactants to alter the wettability of the reservoir in a way that makes it more water-wet. The abandonment stage is the last stage of the field’s life, and it is evaluated by many factors; however, the main point is to determine the economic limit well in advance. The economics of oil and gas production is directly proportional to producible volumes of hydrocarbon, which again is a function of rock wettability.^5−7^ Wettability is a critical parameter in that it controls fluid flow and distribution as well as recovery efficiency in reservoir rocks.^8^ Physically, wettability represents a balance of forces at the interface between three phases, one of which must be solid and defined as the tendency of a fluid to spread or adhere to a solid surface in the presence of other immiscible fluids. It is controlled by the balance between the intermolecular interactions of the adhesive type (liquid to the surface) and cohesive type (liquid to liquid).^9,10^ The wettability parameter of a three-component system (oil–water–rock surface) is classified as follows:^8,11,12^

- Homogeneous wettability: the system has uniform or the same wetting characteristics throughout.
- Preferential wettability: the rock surface is preferentially wet by one of the fluids.
- Neutral or intermediate wettability: the solid surface has no special preference for either oil or water.
- Heterogeneous wettability: rocks with heterogeneous wettability have certain areas preferentially wet by water, while the rest are preferentially oil-wet.
- Mixed wettability: in this type of rock system, the surfaces wet by oil form continuous paths through the larger pores, while the small pores remain water-wet and contain no oil.

The difference in the wettability preferences of different minerals complicates the concept of wettability of reservoir rocks, especially as the rocks have to be classified following the binary-switch classification of either water-wet or oil-wet.^13^ There are several methods for measuring and determining the wettability of reservoir rocks. These methods can be either qualitative or quantitative, each with its associated characteristics and key requirements.^14^

### 1.1 Wettability Measurement Techniques

Traditional approaches for assessing wettability include but are not limited to contact angle measurements using the sessile drop/captive drop procedure, spontaneous imbibition method, Amott–Harvey, U.S. Bureau of Mines (USBM) methods, capillary rise method, and nuclear magnetic resonance (NMR) method. Relative permeability and capillary pressure curves are also used to provide insights into the wettability values of the reservoir rocks.^15−17^ Of all of the wettability measuring methods, the Amott–Harvey and the USBM are the industry standard and most reliable methods for determining wettability.^3^ The A–H index, *I*_AH_, is related to the imbibition characteristics of the rock, and the USBM index is related to the area under the capillary pressure curves. Though these two are internationally accepted standard methods, both are very time-consuming, require a high level of expertise, and require expensive equipment setup.^18^

Another major limitation of the Amott–Harvey index is that a neutral wetting state cannot be measured as either water or oil can be freely imbibed into the rock. Other disadvantages include the long period of time it requires to imbibe and drain fluids out of the core, in addition to the contamination and wettability alteration that the core might be exposed to during the coring process. Similarly, other less preferred wettability measurement processes have pros and cons too.

Dynamic contact angle measurement using a rock piece is another acceptable method, which does not require an elaborate experimental setup or a high level of expertise. However, the contact angle is influenced by surface chemistry heterogeneity and roughness, which is always prevalent in a hydrocarbon reservoir rock. This results in contact angle hysteresis, defined as the difference between the advancing and receding contact angles.^19^ The larger the surface roughness, the larger would be the contact angle hysteresis.^20^ Investigations have proved that even ideal homogeneous solid surfaces exhibit contact angle hysteresis.^21−23^ Thus, it is expected that a high contact angle hysteresis will be exhibited in the case of reservoir rocks with high heterogeneity and roughness. Table 1 captures different established methods for determining wettability along with their merits and demerits.

**Table 1:** Different Capabilities and Features of Wettability Measurement Techniques^a^

technique	quant	qual	time	multiple experiments	temp	res. rock	pressure	expense
Amott	X		10 days	Y	N	Y	N	high
USBM	X		1–2 days	Y	N	Y	N	high
contact angle		X	1–2 h	N	Y	Y	Y	high
NMR	X		10 months	N	Y	Y	Y	high
chromatographic	X		1–2 days	N	Y	Y	Y	high
interfacial tension		X	1 h	N	N	Y	N	med
capillary pressure		X	1 day	N	N	Y	N	med
microscopic		X	1 day	N	N	Y	N	high
FESEM		X	1 day	N	N	Y	N	high
AFM		X	1 day	N	N	Y	N	high

a Time is per sample—approximate time to make the measurement, not including the sample preparation time. Multiple experiments refer to the requirement for multiple experiments to produce a quantitative result. Temp. refers to the ability to make measurements at an elevated temperature. Pressure refers to the ability to make measurements at high pressure. Res. rock refers to the ability to measure rock surface rather than artificial surface. Expense is a qualitative assessment. Y, yes. N, no. X: classifies the technique as being either quantitative or qualitative.

**Figure 1:**
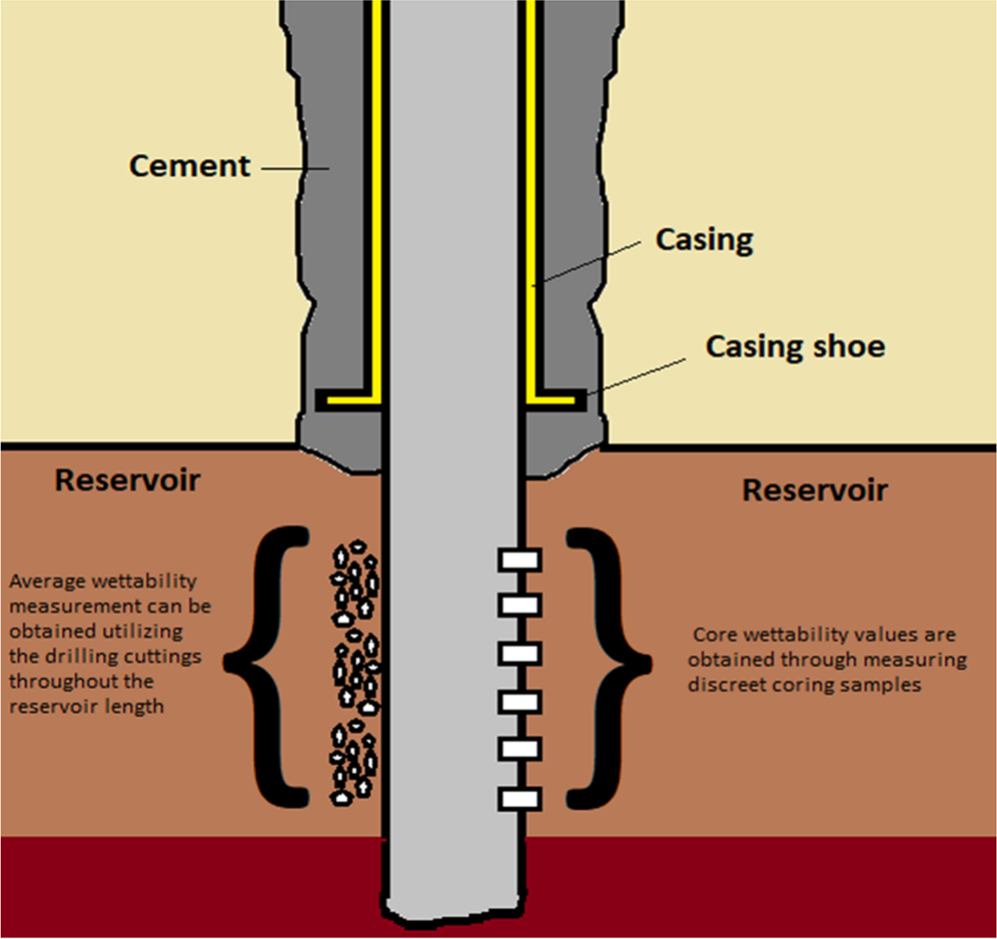
Formation sampling comparison in terms of whole core and unconsolidated rock samples (e.g., drill cuttings).

Notably, all of the methods listed in Table 1 provide wettability values of a tiny portion of the reservoir from which the core samples were obtained. They facilitate point measurements as shown in Figure 1, which cannot provide accurate wettability information on the entire reservoir. Reservoir simulation conducted to predict recovery potential and other important reservoir behaviors can be more accurate if the wettability is measured across the length of the well and not at a few points. This would require wettability measurement of tens of thousands of samples, which is physically impossible and cost-prohibitive. The method presented in this work provides a wettability assessment approach that can use whole core, core fragments, or drill cuttings and provides an average wettability of the reservoir by performing a few simple tests within a short period. The drill cuttings are sampled during the drilling process and offer the opportunity to extract samples from all of the layers of the reservoir, thereby enabling a more accurate determination of the average wettability of the reservoir without having to invest in dedicated coring expeditions.

The floating particle or floatation method was initially considered a qualitative wettability measurement method, where a sample of rock powder is immersed in a representative fluid mix (oil and brine). The wettability is then assessed based on the separation and settlement of the powder particles in each phase.^24^ Most of the rock powder will float in the oil phase if the rock is oil-wet, whereas, in a water-wet system, most of the particles will settle in the water phase. Qualitative estimation of wettability by the floatation method has been known for some time; however, the method is neither established as a quantitative method nor standardized against an industry-accepted standard method. Marmur^25^ used the floatation method to characterize the wettability of monosized spheres of various surface coating materials in an ideal condition. The outcome of this study was an optimization of methanol and ethanol proportion and linking floatability with the particle surface energies. Crawford and Ralston^26^ studied the floatation domain of hydrophobized quartz particles under conditions of known bubble size and relative turbulent velocity and suggested particle size and contact angle domain at which the particle will float. However, their objective was particle separation and not wettability measurement. Film floatation is another technique that can be used to assess particulate wetting characteristics and hydrophobicity. Wetting characteristics of various hydrophobic materials (sulfur, silver iodide, paraffin, wax-coated coal, etc.) measured through film floatation and contact angle methods are in good agreement.^27,28^ Particle floatation studies are also conducted through the powder blasting method wherein the effect of wettability on penetration and floatation behavior of a particle into a liquid is examined.^29^ However, the context and purpose are entirely different from the present objective.

An extensive literature survey shows that the floatation method or floating particle wettability measurement is quick and utilizes minimum and basic tools commonly available in most laboratories. It also uses rock powder instead of the whole core, which opens the door to many opportunities, such as using the cheap and easily available drill cuttings representing the entire reservoir along the good path, whether vertical or deviated. Thus, it can generate a representative and reliable average wettability of the reservoir, which is extremely important for EOR and reservoir simulation studies. Moreover, the floatation method comes in handy when dealing with unconsolidated rocks, where core plugs with good integrity may not be available. The floatation method can be performed under high-pressure and high-temperature conditions to mimic the reservoir environment by fabricating a simple pressure cell with quartz windows. In spite of such possibilities, the floatation method is not yet accepted widely because it is not standardized and lacks quantifiable results. This work presents a method of standardization and quantitative results of reservoir rock wettability from the floatation method. It is envisaged that developing a nonconventional, fast, and easy method for determining reservoir wettability would help both the research field and other reservoir-related studies, especially in resource-constrained locations.

The illustration in Figure 1 shows the different ways of retrieving a formation sample comprising of the drill cuttings and the coring operation.

## 2 Materials and Methods

### 2.1 Materials

#### 2.1.1 Crude Oil

A surface crude oil sample was obtained from one of the Middle East oil fields using an HP cylinder under a nitrogen environment. It was centrifuged at 3000 rpm to remove suspended particles, filtered through a 0.3 micron filter, and stored and later analyzed. The properties of the crude oil sample are given in Table 2, which shows that the oil is rich in acidic and other polar compounds such as resins and asphaltenes.

**Table 2:** Properties of the Crude Oil at Surface Conditions

				SARA analysis (%)			
API gravity	viscosity (cP)	total acid number (mg KOH/g oil)	total base number (mg KOH/g oil)	saturates	aromatics	resins	asphaltenes
40.6	4.28	0.28	0.076	61.75	33.36	3.57	1.31

#### 2.1.2 Dodecane

Laboratory-grade dodecane with more than 99% purity was used as the nonpolar oil phase. Dodecane is used because it is available with a high purity level (99%). It is a linear, paraffin, and an excellent nonpolar solvent or oil alternative, which would help to deduce the effect of polar compounds in crude oils on wettability properties.

#### 2.1.3 Brine

The brine used in this study was 4% KCl, well-settled, decanted, and filtered.

#### 2.1.4 Rock and Core Sample Preparation

Core samples of various rock fabrics with different mineralogies and varying degrees of diagenesis have been collected from four different formations of Middle East fields. The whole cores were used to extract core plugs (used for A–H wettability measurements). The broken pieces were cleaned in the soxhlet extractors within thimbles with toluene until all of the oil and the adsorbed organic compounds were removed. The pieces were further cleaned in soxhlet with methanol to remove inorganic salts. The pieces were then dried, pulverized, and soaked in methanol for 24 h to remove inorganic salts. The samples were then filtered, dried to constant weight, and subjected to sieving through a narrow range of screen meshes. The samples were separated in <25, 25–32, 32–45, 45–53, 53–63, 63–75, 75–90, 90–106, 106–125, and >125 μm size ranges. The size distribution was kept as narrow as possible to achieve maximum accuracy in the final size selection.

The core plugs selected for the Amott–Harvey wettability studies were subjected to the standard cleaning procedure (following the same procedure as the broken pieces), followed by measuring dimensions and determining helium porosity and air permeability. The methodology used for the sample preparations could be found in the literature.^30,31^Table 3 shows the petrophysical properties including length, weight, diameter, porosity, and permeability.

**Table 3:** Properties of the Selected Core Plugs

core #	length (cm)	diameter (cm)	permeability *K* (mD)	porosity (%)
A-1	6.509	3.798	8.45	16.842
B-3	6.551	3.799	6.98	15.835
D-4	6.542	3.795	4.50	17.487
M-7	6.540	3.816	7.38	13.777

## 3 Experimental Methods

### 3.1 Scanning Electron Microscopy–Energy-Dispersive X-ray Analysis (SEM–EDX)

A Hitachi field emission SEM with a Bruker Annular Quad EDX detector is used for the qualitative elemental information of the rock samples. The standard methodologies can be found in Corelab^32^ and other literatures.

### 3.2 X-ray Diffraction (XRD) Analysis

XRD analysis of rock is a standard technique that permits reproducible and accurate calculation of the mineral contents of rocks. The working principle is based on diffraction pattern identification. Details of the analysis method can be seen from the work of Środoń et al.,^33^ and its application on reservoir rock is explained by Muktadir et al.^34^

### 3.3 Amott–Harvey Wettability Study

Amott–Harvey wettability is considered the industry’s most reliable wettability measurement method. This method involves five different stages, which were conducted as (1) first forced drainage and (2) spontaneous imbibition, first forced imbibition, spontaneous drainage, and finally secondary forced drainage. Forced drainage and imbibition were conducted by using Corelab ACES-300 automated ultracentrifuge equipment, while spontaneous drainage and imbibition were performed using Amott cells. The idea of ultracentrifuge was to apply centrifugal force to displace a liquid from saturated core samples under different RPMs. Ultracentrifuge speed was increased in steps till *S*_or_ or *S*_wirr_ conditions were established. The maximum required speed for this purpose was 11 000 rpm; however, the rotor was run up to 12 000 rpm. Capillary pressure and Amott wettability index were calculated after analyzing the produced data. Amott cells could directly give the volumes of water or oil spontaneously imbibed into the core plugs. The Amott–Harvey Index was calculated as follows. The water wettability index, *I*_w_, the oil wettability index, *I*_o_, and the Amott–Harvey index are defined as
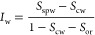
1
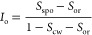
2
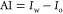
3where *S*_cw_ is the connate water saturation, *S*_or_ is the residual oil saturation, *S*_spw_ is the water saturation at zero capillary pressure, and *S*_spo_ is the oil saturation at zero capillary pressure.

AI ranges between +1 and −1, where +1 represents the most strongly water-wet and −1 represents the most strongly oil-wet characteristics. The detailed classifications are as follows: an AI value between +1 and +0.3 implies water-wet, an AI value from +0.3 to 0 indicates a weakly water-wet system, while an AI value between −1 and −0.3 means oil-wet; when it falls in the range of −0.3 to 0, it indicates a weakly oil-wet rock.^12,35^

### 3.4 Floating Particle Experiment

From some preliminary studies, it is found that the size of rock particles greater than 125 μm and less than 25 μm is not suitable for floatation studies because of either excessive gravity settling or buoyancy effects, respectively, which may produce erroneous results.

The optimum particle size was determined based on the best-fit selection that reflects the actual wettability value that matches the wettability obtained through the Amott–Harvey tests. For each sample, the recommended particle size was determined separately.^36^ The steps followed for floatation experiments are:

1. KCl brine (4%) was prepared, and each separating funnel was filled with 50 mL of brine.
2. One gram of the pulverized sample was weighed and poured into a separating funnel containing the brine. The separating funnel was closed and then shaken vigorously to drive out any air that could have been attached to the powder samples.
3. Fifty milliliters of oil was then added to the separating funnel and shaken vigorously to allow the constituents to mix properly.
4. The particles were distributed among the brine and oil phases depending upon their preference. Some particles could be seen at the bottom, some floating while some were at the oil–water interface.
5. The setup was allowed to stand undisturbed for 24 h, so particles could undergo proper distribution in either phase, depending on their preference.
6. The particles in the water phase were collected on filter paper by draining out the water through the opening in the separating funnel. The initial dry weight of filter paper without particles was noted down.
7. Care was taken during draining to prevent any particles from the oil phase from escaping to the water phase.
8. The particles collected were cleaned with toluene and then with methanol to remove any oil that could have been on the particles and could add up to the rock weight.
9. The particles were then dried in the oven for an hour and then weighed along with filter paper.
10. The particles in the water phase were obtained by subtracting the reading of dry filter paper containing particles and dry filter paper.
11. The process was repeated 3 times for each particle size and each fluid combination, viz. brine/dodecane, brine/crude, and brine/crude–dodecane (50:50 mixtures), and the average values were reported.
12. Similar studies were conducted for 45–53, 53–63, 63–75, 75–90, 90–106, and 106–125 μm sized samples prepared from A-1, B-3, D-4, and M-7 sample rocks.

**Figure 2:**
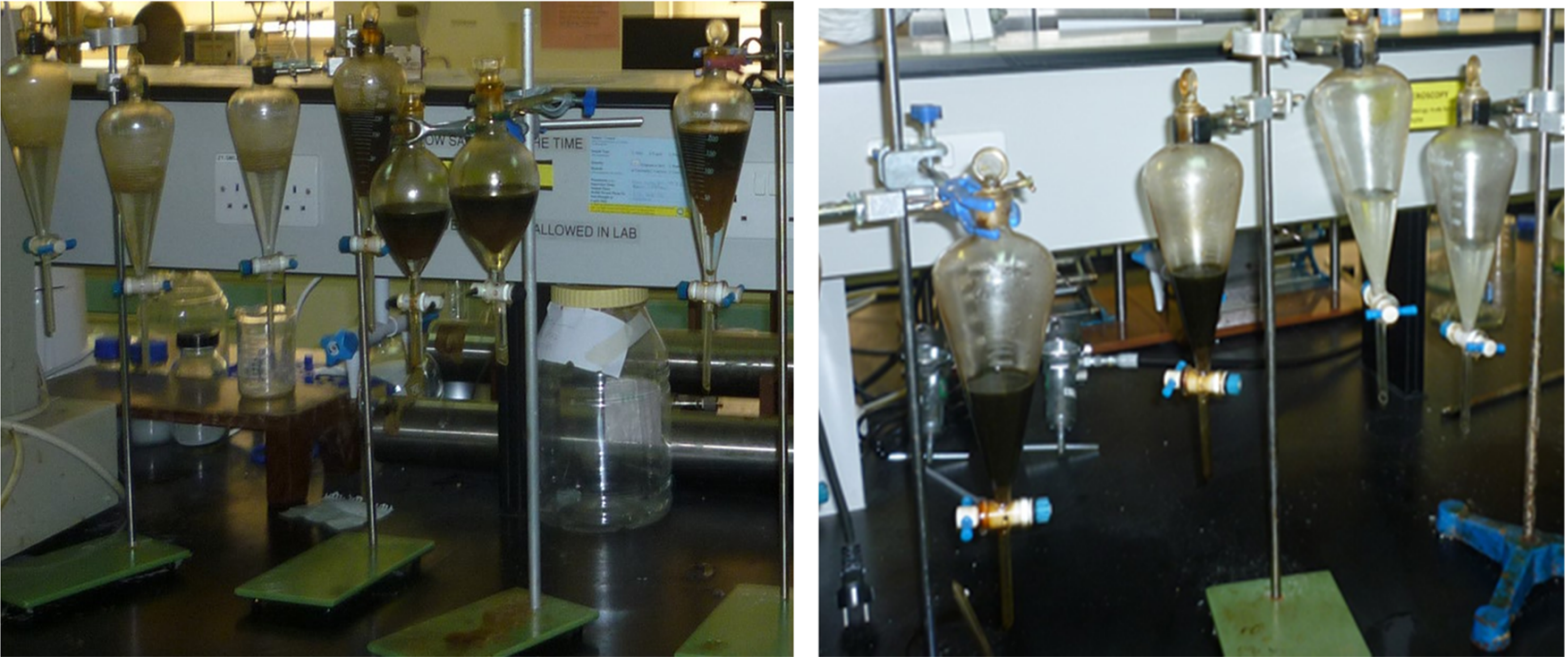
Floatation experiment setup before draining (left) and after draining (right).

Figure 2 shows the experimental setup for the floatation experiments.

## 4 Results

### 4.1 SEM–EDX and XRD-Based Mineralogical Analysis of the Samples

**Figure 3:**
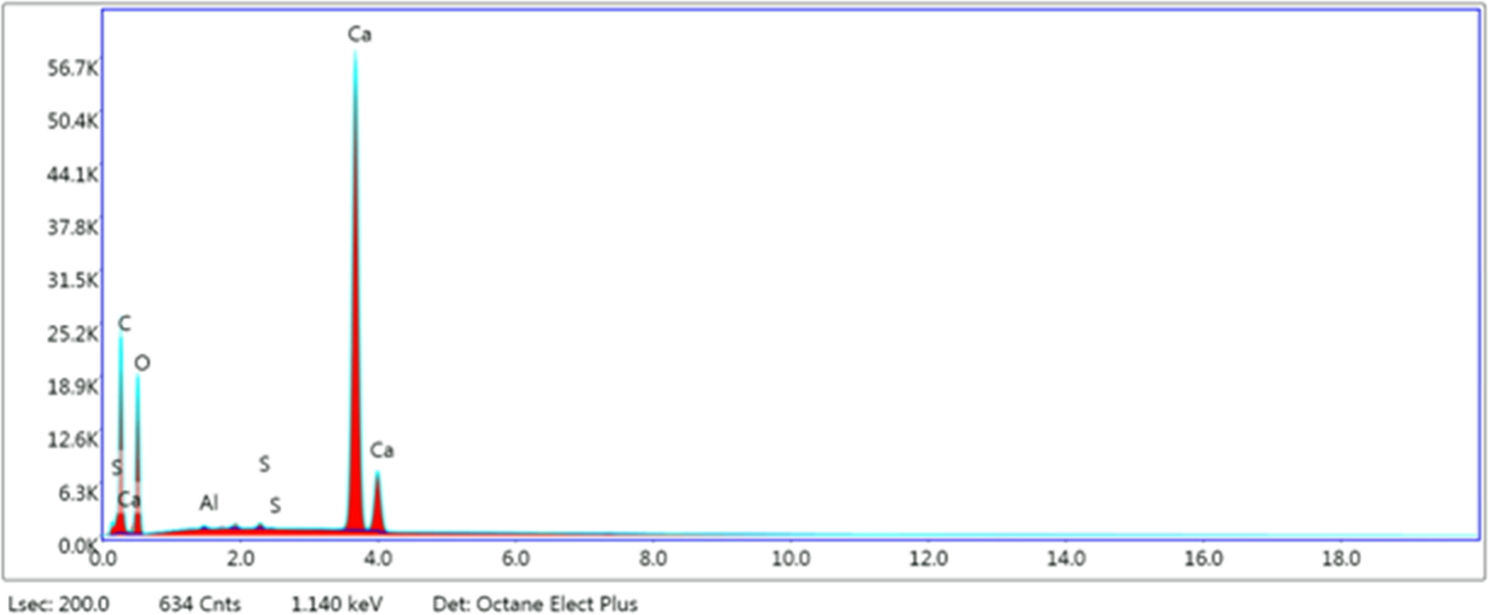
Elemental composition of sample M-7.

Scanning electron microscopy (SEM) with energy-dispersive X-ray analysis (EDX) provides elemental identification and quantitative compositional information.^37^ Result of the EDX analysis for sample M-7 is given in Figure 3. For the other three samples, the SEM results can be seen in the appendix A (Figures A-1–A-3). The chemical compositions of the four rock samples are shown in Table 4.

**Table 4:** Comparative Analysis of the Major Elements of the Rock Samples

	wt %				atom %			
element	M-7	B-3	D-4	A-1	M-7	B-3	D-4	A-1
Ca	98.72	93.13	88.75	95.05	98.33	90.84	85.71	92.67
Mg	0.00	1.21	0.24	3.21		1.78	0.00	5.08
Al	0.55	1.30	0.98	0.44	0.78	2.04	1.43	0.59
Si	0.00	1.30	1.47	0.49		1.78	2.14	0.71
S	0.73	3.06	8.56	0.83	0.89	3.56	10.71	0.95

#### 4.1.1 SEM–EDX Result Analysis

It should be mentioned here that the carbon and oxygen values were not included in the analysis because their source is from the adhesive tape upon which the rock powders were spread during the experiments.

#### 4.1.2 X-ray Diffraction Result Analysis

**Figure 4:**
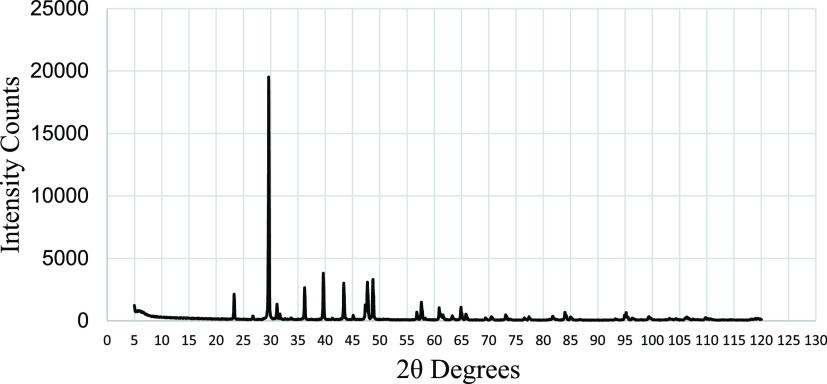
XRD analysis of the M-7 sample.

XRD analysis is used to identify the crystalline phases present in a material and hence identify the mineralogy of the rock. The acquired 2θ values are compared with a reference database to identify the test sample’s phases. The result of the XRD analysis for sample M-7 is given in Figure 4. The XRD plots for the other three samples can be seen in the appendix B (Figures B-1–B-3). The mineralogical analysis of the samples is presented in Table 5.

**Table 5:** Mineralogical Analysis of the Rock Samples

	quantitative rock mineralogy by X-ray diffraction (XRD)								
	percentage of whole-rock								
sample no.	calcite	dolomite	halite	anhydrite	albite	pyrite	quartz	clay	total
M-7	99.4	0	0	0.3	0	0	0	0.3	100
B-3	94.5	1.3	0	2.5	0	0	0.9	0.8	100
D-4	85.8	0.4	0.3	10.2	0.4	1.2	1.0	0.7	100
A-1	94.2	4.8	0	0.3	0	0	0.3	0.4	100

### 4.2 Amott–Harvey Wettability Results

**Figure 5:**
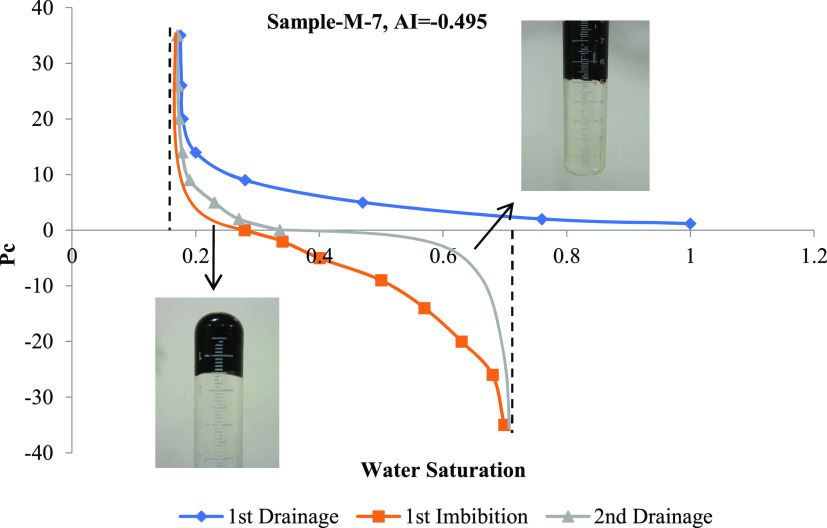
Drainage and imbibition plot and indices of the core sample M-7.

The Amott–Harvey wettability index for the four core plugs was calculated based on the measured drainage and imbibition saturations against capillary pressure values. One sample plot (Figure 5), the stepwise calculation method using relevant equations (eqs 1–3), and the resulting A–H index are presented in Table 6. Table 7 shows the Amott–Harvey index and possible wettability characteristics of all four rock samples.

**Table 6:** Results of the Saturation Parameters of M-7 from the Saturation vs Capillary Pressure Test

	remaining oil (cm^3^)	remaining water (cm^3^)	fluid saturation
2nd forced drainage	11.802	2.497	*S*_cw_ = 0.175
spontaneous imbibition	10.303	3.996	*S*_spw_ = 0.279
forced imbibition	4.303	9.996	*S*_or_ = 0.300
spontaneous drainage	9.503	4.796	*S*_spo_ = 0.664

**Table 7:** Amott–Harvey Wettability Index of the Four Rock Samples

sample no.	*I*_w_	*I*_o_	A–H wettability index	remarks
M-7	0.198	0.693	–0.495	oil-wet
B-3	0.211	0.629	–0.418	oil-wet
D-4	0.158	0.543	–0.385	oil-wet
A-1	0.174	0.509	–0.335	oil-wet

Using eq 1, we get 

Using eq 2, we get 

And using eq 3, we get AI = *I*_w_ – *I*_o_ = 0.198 – 0.693 = −0.495.

From Table 7, it can be seen that all of the rock samples are oil-wet. In comparison, the M-7 sample has the highest oil-wetting property and the A-1 is the least oil-wetting rock.

### 4.3 Results of Floatation Experiment

**Figure 6:**
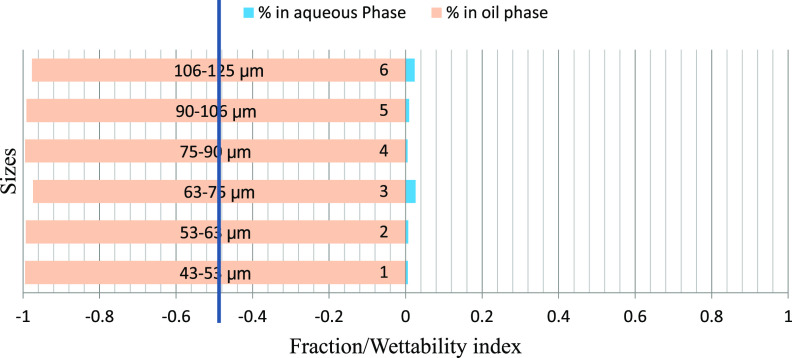
M-7/brine/crude oil fraction distribution.

**Figure 7:**
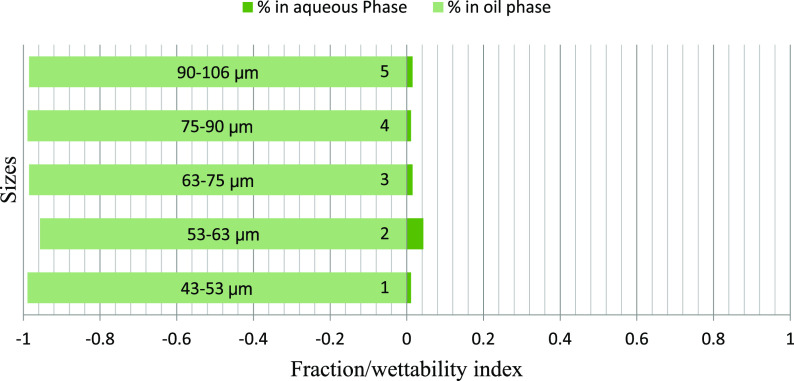
M-7/brine/50:50 crude oil–dodecane fraction distribution.

**Figure 8:**
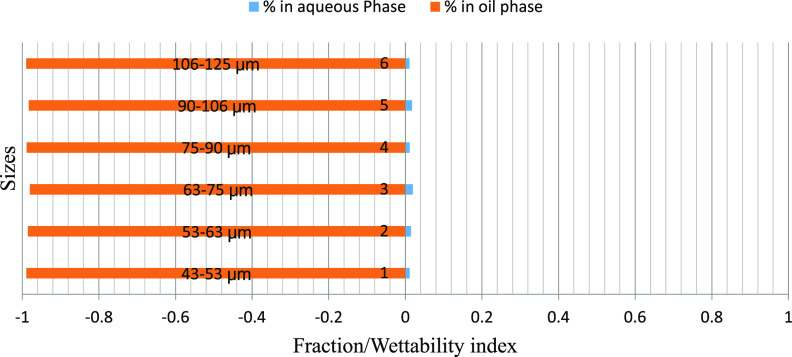
M-7/brine/dodecane fraction distribution.

**Figure 9:**
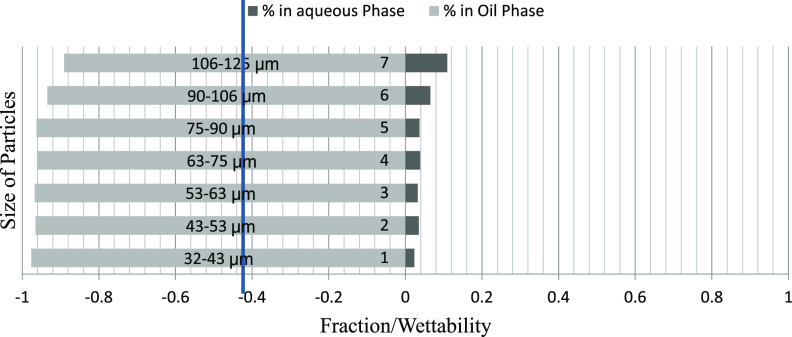
B-3/brine/crude fraction distribution.

**Figure 10:**
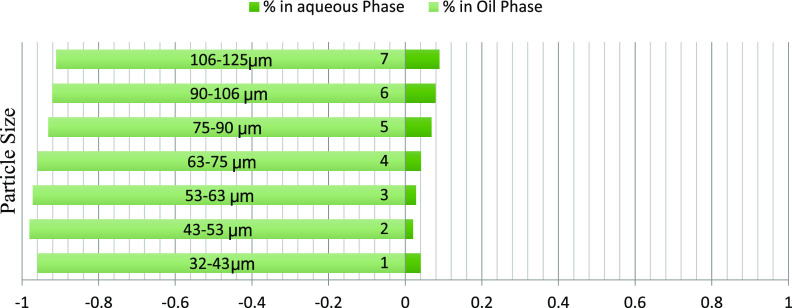
B-3/brine/50:50 crude–dodecane fraction distribution.

**Figure 11:**
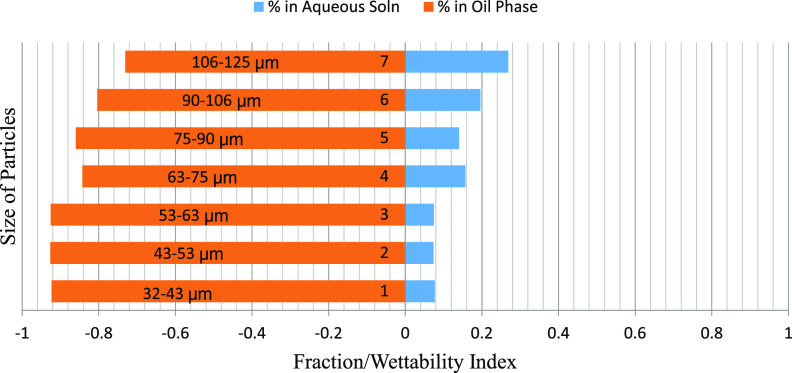
B-3/brine/dodecane fraction distribution.

The mass fraction of particles obtained in the water and oil phases using 4% of KCl brine and oils of different polarities with different particle size ranges is depicted graphically. Figures 6–8 represent the particle fraction distribution of the M-7 sample. Figures 9–11 represent the same for the B-3 sample. The fraction distribution of D-4 and A-1 samples, which are similar to that of B-3, is shown in Appendices C Figures C-1–C-6. The compiled results are presented in Table 8. In the present case, the crude oil has the highest polar fraction, followed by 50:50 crude/dodecane (due to dilution) followed by dodecane, a nonpolar hydrocarbon. The fractional mass distribution is presented on a scale of +1 to −1 with the mid- of the scale at zero. If all of the particles are in the water phase, they are placed between +1 and 0 scale and considered 100% water-wetting. Similarly, if all of the particles are in the oil phase, it is placed between 0 and −1 scale and considered 100% oil-wetting. It can be generally observed from the figures that the fraction in the water phase increases with the increase of the size range of the particles, showing the dominance of gravity over buoyancy. Another general observation is that the maximum fraction of rock powder in the oil phase was obtained when crude oil was used as the oil phase, and the minimum fraction was obtained when dodecane was in the oil phase. This can be attributed to crude oil containing predominantly acidic polar fractions (evident from TAN, and SARA analysis given in Table 2). It is well known that polar compounds have profound effects on rock wettability in a carbonate formation, particularly the acidic components, which help the oil blobs adhere to the positively charged calcite surface, rendering them oil-wet.^38,39^ Dodecane being void of any polarity is expected to have a lesser tendency to attach to the calcite surface. The 50:50 crude/dodecane results were in between the two extremes. This observation is supported by the core flood results and the wettability characteristics reported by Puntervold et al.^40^ They found that the carbonate rocks have a significant preference for the adsorption of acidic components of crude over basic fractions, and the minerals present on the rock surface profoundly influence the adsorption of polar organic compounds and wettability at the equilibrium state.

**Table 8:** Floatation Sample Experimental Results

	fluid phases					
	CO_100_–Brine		CO_50_:DD_50_–Brine		DD_100_–Brine	
rock sample	% in OP	% in BP	% in OP	% in BP	% in OP	% in BP
M-7 (43–53 μm)	99.45	0.55			98.90	1.10
M-7 (53–63 μm)	99.30	0.70	98.90	1.10	98.55	1.45
M-7 (63–75 μm)	97.40	2.60	95.67	4.33	98.08	1.95
M-7 (75–90 μm)	99.48	0.52	98.85	1.15	98.85	1.15
M-7 (90–106 μm)	99.10	0.90	98.93	1.07	98.28	1.72
M-7 (106–125 μm)	97.65	2.35	98.50	1.50	98.95	1.05
D-4 (32–43 μm)	97.90	2.10	98.90	1.10	86.17	13.83
D-4 (43–53 μm)	97.90	2.10	97.00	3.00	77.6	22.40
D-4 (53–63 μm)	95.97	4.03	95.27	4.73	75.87	24.13
D-4 (63–75 μm)	90.53	9.47	89.13	10.87	52.20	47.80
D-4 (75–90 μm)	88.77	11.23	92.43	7.57	59.00	41.00
D-4 (90–106 μm)	85.77	14.23	89.90	10.10	52.45	47.55
D-4 (106–125 μm)	84.80	15.20	89.70	10.30	46.90	53.10
B-3 (32–43 μm)	97.63	2.37	96.03	3.97	91.03	8.97
B-3 (43–53 μm)	97.30	2.70	98.03	1.97	93.53	6.47
B-3 (53–63 μm)	97.13	2.87	97.20	2.80	92.53	7.47
B-3 (63–75 μm)	96.55	3.45	96.67	3.33	84.30	15.70
B-3 (75–90 μm)	97.07	2.93	95.13	4.87	87.17	12.83
B-3 (90–106 μm)	94.60	5.40	93.65	6.35	81.87	18.13
B-3 (106–125 μm)	90.40	9.60	91.05	8.95	75.27	24.73
A-1 (32–43 μm)	98.23	1.77	94.93	5.07	73.47	26.53
A-1 (43–53 μm)	94.23	5.77	94.10	5.90	59.80	40.20
A-1 (53–63 μm)	95.73	4.27	90.30	9.70	61.03	38.07
A-1 (63–75 μm)	87.20	12.80	90.05	9,95	32.07	67.93
A-1 (75–90 μm)	84.40	15.60	89.00	11.00	38.03	61.97
A-1 (90–106 μm)	71.67	28.33	72.33	27.67	38.50	61.50

Buoyancy and density of particles are also to be considered in floatation wettability results as they would impact the ratio of particles in oil and water phases. The viscosity of oil would play a role in this regard as it would enhance the buoyancy effect. The diluted crude oil and dodecane reduced oil polarity and the oil viscosity, resulting in a higher percentage of rock particles sinking into the water phase. This would apparently display higher water wetness in the floatation experiment, evident from the three sets of experiments conducted with three different oil types. These observations indicate that crude oil should be used in floatation experiments instead of any synthetic oil for a valid conclusion.

The overall results strongly indicate that the particle size clearly affects the particle distribution in the two phases. This observation was the motivating factor for a deeper-level study to establish the optimal particle size, which would generate a reliable fractional distribution pattern and help in deducing the quantitative wettability value. For this purpose, the A–H wettability index number from Table 7 is placed on the fractional distribution plots as a vertical bar considering the same +1 to −1 scale considered for A–H indexing. Thereafter, the particle size range, which balances the AH index (i.e., 50% of the particles are on the right side and 50% on the left side of the AH index bar), is considered the most appropriate particle size range. A–H index bars are placed on the crude–brine plots only as the A–H experiments were conducted only with the crude oil–brine systems. A–H wettability measurement for crude–dodecane and dodecane systems could not be conducted due to facility constraints. The particles obtained in the brine and oil phases after using different fluid combinations (brine/dodecane, brine/crude oil, brine/50:50 crude/dodecane) and with different particle size distributions for the rock sample M-7 are given in Figures 6–8. The fraction of particles obtained in all of the size ranges clearly indicates a rock’s oil-wetting characteristic. Increasing the sizes of the particles does not seem to have much impact on their fractional distribution (within the measured particle size range). Similar results were also obtained for reduced polarity and nonpolar oils. In all three oils/brine scenarios, more than 98% of the particles adhered and float into the oil phase, defying gravity irrespective of particle size. The A–H index of this sample is −0.495, which indicates oil-wetting characteristics of the rock. Thus, it can be concluded that if the rock is oil-wetting, the particle size (within the range of investigation) has little significance and the oil’s polarity plays a less significant role.

The fraction of particles obtained in the brine and oil phases after using oils of different polarities and different particle size ranges for rock samples B-3, D-4, and A-1 demonstrated an exciting trend. The fraction obtained in the water phase increases with particle size, with a few exceptions. Among different polarity oils, the crude oil–brine system shows minimum particle fraction into the water phase, indicating the effect of the polarity of oil on the particle wettability. The fractions obtained in the 50:50 crude–dodecane were between the two extremes. These results further confirm the effect of size and rock/oil/brine interaction on the distribution of particles between phases. The A–H index bars show that for the B-3, D-4, and A-1 samples, the 50:50 particle distribution occurs on the particle sizes of 63–75 μm and 75–90 μm ranges. The smaller particles have an increasingly higher preference toward oil due to the buoyancy advantage, and the larger particles sink to brine due to the increased gravity effect. Thus, it can be concluded that for weakly oil-wet rocks, the appropriate particle size may range anywhere between 63 and 90 μm, which will well represent the ideal particle size range for conducting floating particle wettability measurement, and the value is expected to match with the A–H wettability index.

The plots representing the particle fraction distribution in brine/50:50 crude/dodecane and brine/dodecane show an increasing trend of water wettability. As the crude oil is diluted and oil polarity is reduced, more particles change their behavior from oil-wetting to water-wetting due to the reasons explained earlier. These observations prove the validity of the floating particle method and make it suitable for nonpolar systems also. Though the A–H study could not be conducted with dodecane and dodecane-diluted oil, the trend is encouraging and matches the expectations.

### 4.4 Floatation Experimental Setup Results

Table 8 shows the results of the floatation experiments conducted on the rock samples as listed in Table 3.

## 5 Discussion

The objective of this research was to establish an easy and reliable wettability measurement method, focusing on the floating particle method. The goal was to develop a more effortless and cheaper method for measuring wettability without requiring expensive lab equipment and highly skilled personnel. Rock samples from various formations of Abu Dhabi were collected, processed, powdered, cleaned, and finally sieved to different sizes. The rock samples were characterized with the help of SEM–EDX for elemental analysis and powder XRD method to investigate their mineral compositions. These analysis results are given in Tables 4 and 5. The literature shows that dolomites generally display higher water wetness compared to calcite.^30,41,42^ Also, it is claimed that water wetness increases with sulfate concentration because, all other things being equal, sulfate will sorb onto and locally reverse the charge on the calcite surface to decrease oil adhesion.^43,44^

Accordingly, the M-7 sample, which is composed of 99.4% calcite, is expected to show the highest oil-wet characteristic followed by B-3. Samples D-4 and A-1 have a significant portion of dolomite, anhydrite, clays, and quartz. Because of the presence of these preferentially water-wet materials, these rocks are expected to show lower oil-wet characteristics. Amott–Harvey wettability studies conducted to establish a wettability benchmark support the above assumptions. Although known for qualitative measurement, the floating particle method is not very popular because it lacks the ability to provide quantitative wettability values. This study’s most important aspect was establishing the parameter required to make it a quantitative method, which is “the appropriate and dependable particle size range”. If larger than the optimum particle size is used, the particles will sink because of the gravity effect. Similarly, if the particles are too small, they will tend to float and remain in the oil phase, which can be correlated to Stoke’s law. The distribution of particles in the water and oil phases shows a clear trend based on their wettability measured through the standard A–H method. The most oil-wet sample shows almost all of the particles in the oil phase and their midpoint match with the A–H index. This experiment suggests that if the rock is highly oil-wet, particle size (within the studied range) has little or no impact on particle distribution. The lesser oil-wet rock samples also show a good matching with the A–H index when they are placed on the particle distribution chart. The midpoint analysis shows that a 63–90 μm particle size will be ideal for floating particle wettability measurements, as these are the size range whose midpoint coincides with the A–H index.

Another general observation is that the maximum fraction of rock powder in the oil phase was obtained when crude oil was used as the oil phase and the minimum fraction was obtained when dodecane was in the oil phase. This can be attributed to crude oil containing predominantly acidic polar fractions (evident from TAN, and SARA analysis given in Table 2).

In spite of the matching behavior between the A–H wettability index and the floating particle fractional distribution behavior, and the possible benefits of the floatation method, there are a few concerns that must be addressed. According to Rücker et al.,^45^ core-scale wetting behavior strongly depends on the surface area coverage by oil, which is controlled by the surface structure of the grain surfaces and the capillary pressure applied during saturation. Once the rock is pulverized, it loses its texture, pore, and original surface roughness properties. Further investigation on the microscopic scale is ongoing to investigate these aspects to further strengthen the floatation wettability measurement approach. Several other challenges and shortcomings of the floatation method also need to be considered, such as the aging of rock sample, preference of fluid addition, and oil/brine-to-rock powder ratio, which may impact the wettability results.^46^ The type and concentration of acidic compounds in the crude play a major role in wettability characteristics.^46−48^ Thus, using reservoir crude in the experiment is essential to draw a valid conclusion.^49−52^ Others demonstrated the effect of temperature on wettability through floatation experiments and suggested that the static reservoir temperature should be considered for this study. As a way forward, we are testing HT–HP separating funnels with viewing windows to conduct high reservoir temperature samples. In conclusion, it is not possible to standardize the floatation process universally at this stage unless much more data is generated; however, for a particular formation, the standardization and validation could be easy and useful.

## 6 Conclusions

This article proposes a quantitative approach to calculating rock wettability through the floatation method, whose values agree with the Amott–Harvey results.

Though the floatation method is known for some time, some inherent limitations are observed in this approach, including the fact that the result and behavior of the floating and sinking of particles are highly affected by the particle size selection. For example, if the particles were very large, the dominant behavior would be the settling or sinking of the particles due to the gravity effect. On the other hand, if the particles were too fine, the buoyancy forces would cause them to float in the oil phase (the lighter phase). In both cases, the behavior of the particles does not truly reflect the wettability. Hence, choosing a suitable particle size range and standardizing against a universally accepted method is significant. Amott–Harvey method is considered for standardization of the floatation method, showing a good match with Amott–Harvey wettability results and helping to select suitable particle size ranges.

The most important aspect of this study is that, once the particle size is optimized, this method can be a reliable and rapid wettability investigation method and can provide the average wettability of a section of hydrocarbon reservoir through the use of easily available drill cuttings.

Since floatation wettability results would be affected by a variety of rock–fluid parameters, such as density, viscosity, salinity, and temperature, the particle size selection cannot possibly be generalized; however, once the particle size is standardized for a particular formation condition, the same size range could be used for other samples having similar properties.

### 6.1 Way Forward

The authors are in the process of devising visual cells to conduct floatation experiments at HT–HP conditions close to the reservoir conditions. The effect of oil viscosity, rock density, and formation water salinity will also be investigated. In addition, the impact of rock pulverization, resulting from the destruction of pore structure and surface roughness, is under investigation through microscopic methods such as micro-CT and AFM.

## Figures and Tables

**Figure 12:**
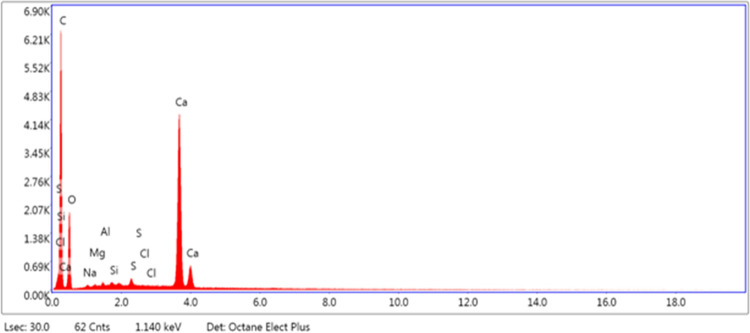
Elemental composition of sample B-3.

**Figure 13:**
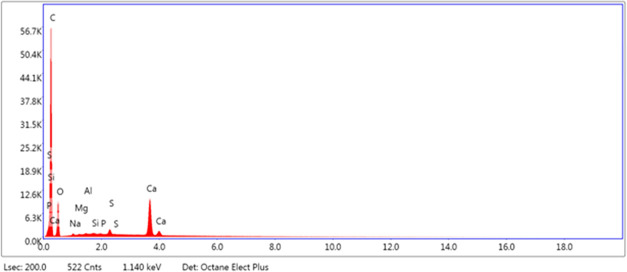
Elemental composition of sample D-4.

**Figure 14:**
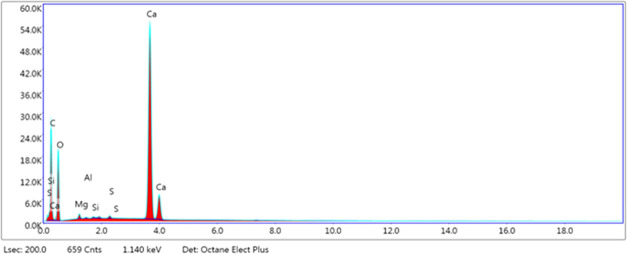
Elemental composition of sample A-1.

**Figure 15:**
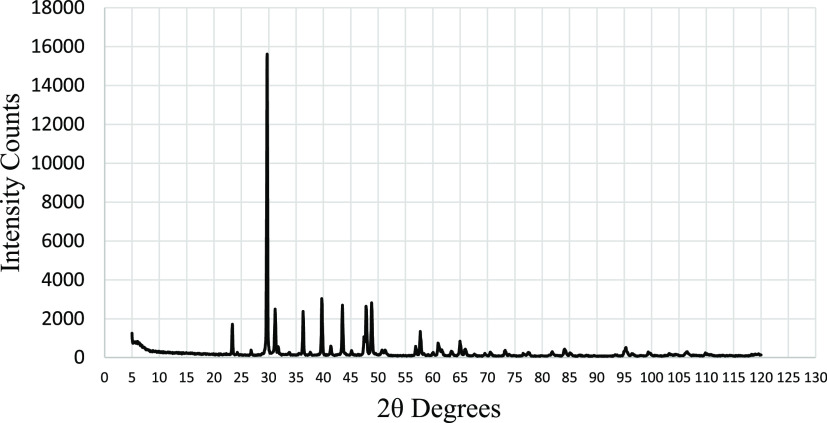
XRD analysis of the B-3 sample.

**Figure 16:**
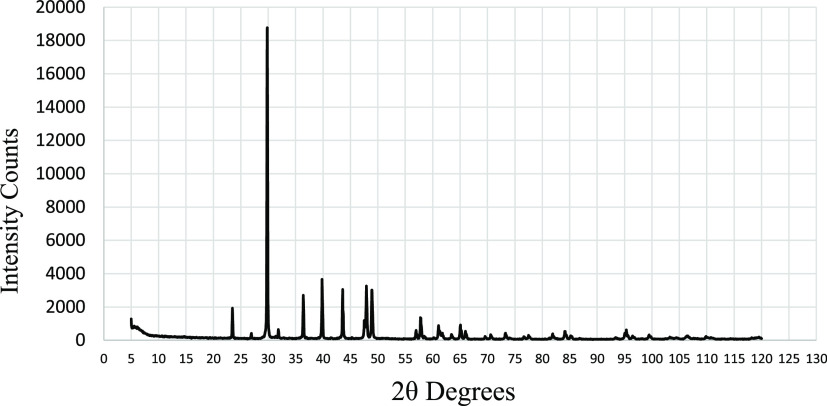
XRD analysis of the D-4 sample.

**Figure 17:**
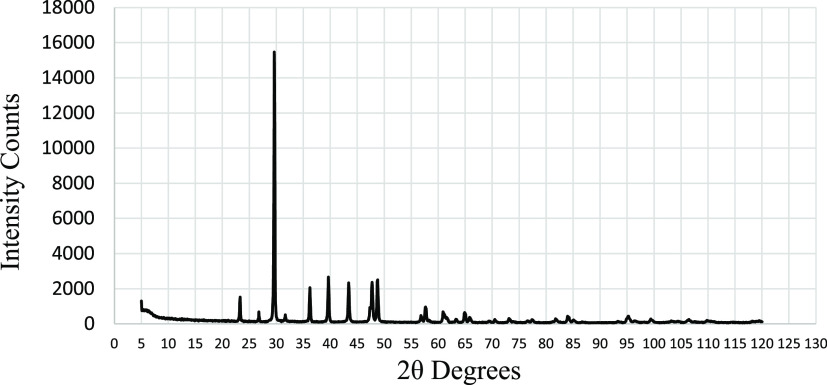
XRD analysis of the A-1 sample.

**Figure 18:**
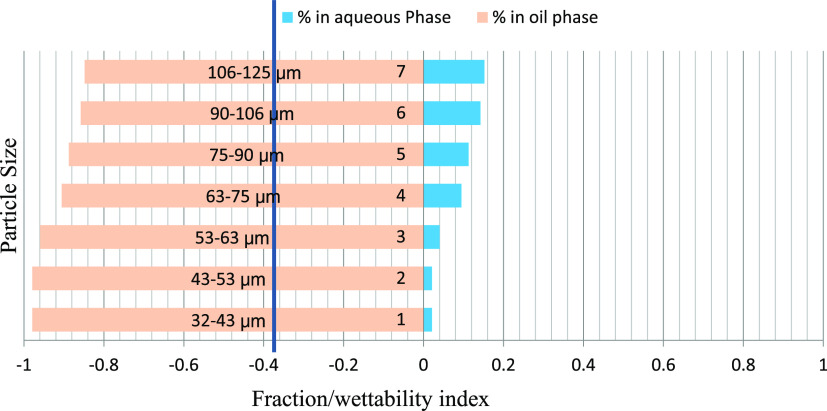
D-4/brine/crude oil fraction distribution.

**Figure 19:**
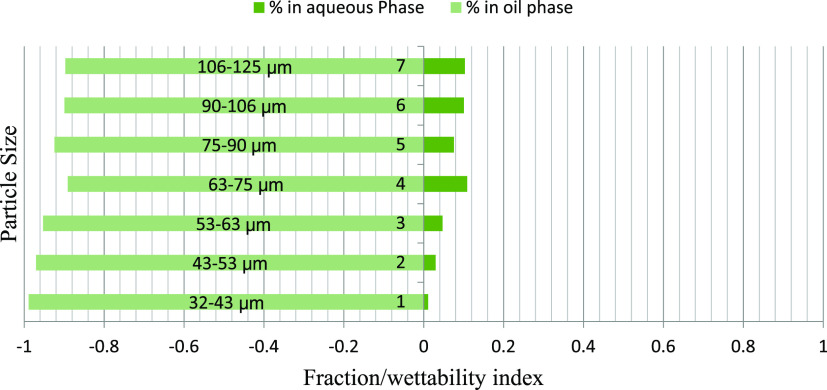
D-4/brine/50-50 crude oil-dodecane fraction distribution.

**Figure 20:**
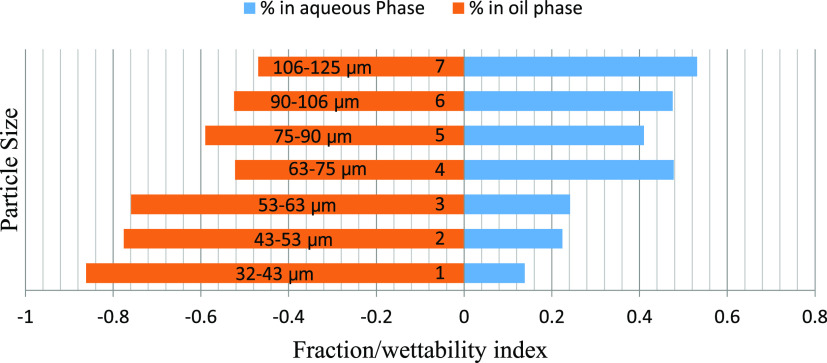
D-4/brine/dodecane fraction distribution.

**Figure 21:**
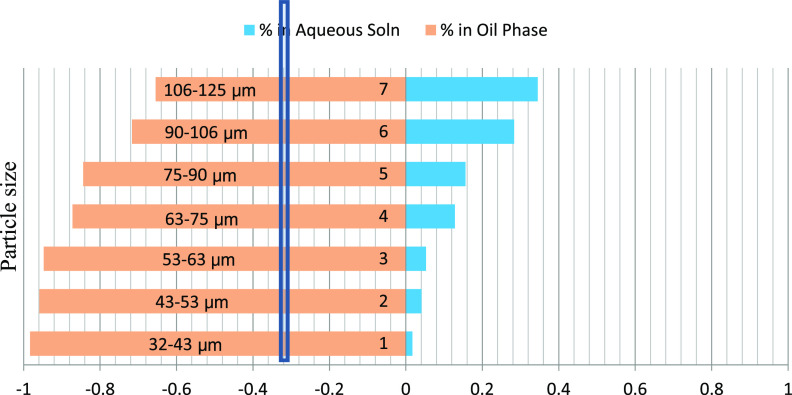
A-1/brine/crude oil fraction distribution.

**Figure 22:**
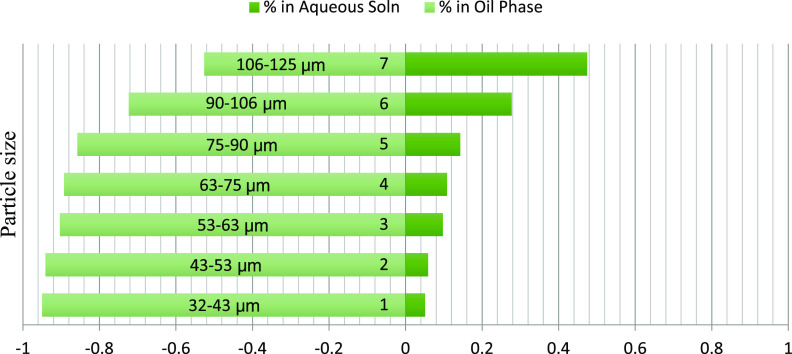
A-1 brine/50-50 crude-dodecane fraction distribution.

**Figure 23:**
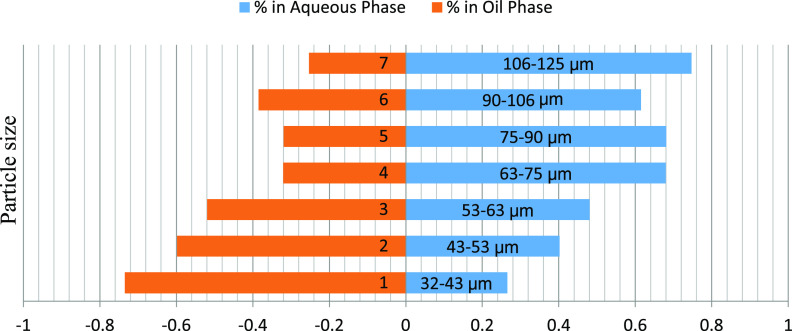
A-1/brine/dodecane fraction distribution.

## Appendix-A

Figures 12A1–14A3

## Appendix-B

Figures 15B1–17B3

## Appendix-C

Figures 18C1–23C6
